# Correction: Rare event detection by progressive clustering undersampling

**DOI:** 10.1371/journal.pone.0352210

**Published:** 2026-06-22

**Authors:** The PLOS One Staff

The images for S1 Fig, S2 Fig, and S3 Fig are incorrect. The image that appears as S1 Fig should be S3 Fig, the image that appears as S2 Fig should be S1 Fig, and the image that appears as S3 Fig should be S2 Fig. Please view the correct S1 Fig, S2 Fig, and S3 Fig below.

The publisher apologizes for the errors.

## Supporting information

S1 Fig Another possible path for PCU process. The final result doesn’t change much.
(PNG)

S2 Fig Evaluation scores for different models measured from Iris test set. Grey models are supervised classifiers trained directly without resampling. Orange models represent KNN executed after different resampling methods. KNN without resampling is sufficient to achieve the desired results for a simple dataset.
(PNG)

S3 Fig Study flow and predictive models generated using various combinations of resampling and classifier techniques. SOTA resampling methods, along with the newly introduced PCU, were evaluated on multiple datasets. The PCU workflow integrates clustering alternated with classification steps to produce Semi-Guided and Fully-Guided decision boundaries.
(PNG)
