# Supplementary material for: Rare event detection by progressive clustering undersampling
Source: PLoS One. 2026 Jan 30;21(1):e0340758. doi: 10.1371/journal.pone.0340758 (PMC12858060; doi:10.1371/journal.pone.0340758)
Supplement: Attachment — Submitted filename: Responses to reviewers.docx [file pone.0340758.s005.docx]

Responses to Reviewers

Dear Editor,
I would like to sincerely thank you and the reviewers for your valuable time, insightful comments, and constructive suggestions and modifications. Your feedback has been instrumental in improving the clarity and quality of this manuscript. We have carefully addressed all points raised, and believe these revisions have greatly enhanced the overall writing.

All additions or changes in the marked-up copy appear in red, while deletions are shown with strikethrough. Answers here include searchable keywords, written in italics.

Reviewer One:
1. Need to add subsection for more explanation about the limitation, challenges, contribution and structure of the article.

Performed in subsec. 5.1.
structure of the article :
‘*The rest of the paper is structured ..’*

2. It is better to add table summarizing the x-studies showing the advantages and disadvantages.

I definitely agree with you that summarizing the related studies in a table would be better. However, we chose to keep the comparison within the text rather than summarizing it because of limitations on the number of tables and pages. We reserved those for the methodology description and results.
3. A detailed analysis of the limitations and potential failure scenarios of the proposed model is missing.

Detailed in subsec. 5.1.
4. Some information about datasets and experiment setup is needed.

Subsec. 3.1 (one page) describes the petroleum dataset. while the second dataset was described in:
*‘The two moons dataset is a synthetic, ….’*
please refer whether a specific information is missing so we can modify or enhance writing.

experiment setup description is enhanced in subsec. 5.2.
5. Additional comparative analysis, around computational requirements, cost and robustness of the model with other SOTA methods.

*‘Three evaluation metrics were assessed on two datasets’…*

Regarding the computational aspect, here is our perspective:

PCU simply integrates one or more common supervised and unsupervised algorithms to undersample the training set. The computational complexity is tied to the specific algorithms used, with their exact complexity functions typically outside the scope of this work and usually obtainable from the foundational algorithm papers.

In general, undersampling methods may require time to reduce the number of samples, but they offer clear advantages during model building compared with oversampling techniques.

6. Provide quantitative remarks of the impact of the proposed method in the abstract and conclusion.

The quantitative results were found to be too extensive to include in the abstract due to character limitations. Listing the scores of PCU branches compared to the next best resampling methods for both datasets, in terms of F-score and precision, would exceed the allowable length. Therefore, we instead summarized these findings in the conclusion:

*‘When using the KNN classifier with various resampling …’*
7. References needed to be updated (24/25).

Updated with related articles.

Reviewer Two

Methodological Clarity and Reproducibility

The PCU algorithm is interesting but described in a largely conceptual way. The pseudocode in Algorithm 1 lacks specific implementation details such as:

- How clustering algorithms are selected or switched between stages.

A list of user-defined algorithms required to run the PCU and can be used in all stages for simple implementation. PCU compares between clusters and select the unique one regardless of its origin:

‘PCU is not tied to a specific unsupervised algorithm …’,

Criteria used for determining the “rupture” point.

This was answered in:

‘*until the minority instances can* …’	to ‘*formation of the last cluster’.
in addition, clearly stated in the added subsec. 5.2*

*We Also:*

- Explained farther the pseudo-code (Algorithm 1).

- Differentiated between customized version of PCU and automated one.

- Parameter tuning strategies for clustering algorithms.

We focused more on testing parameters of resampling algorithms, since the study’s main goal is to compare different resampling methods. Tuning unsupervised parameters would be extensive and could lead to a huge number of trials when combined to other options. This is illustrated in:
‘Clustering algorithms were employed in the baseline setup …’.

Please expand this section with more formal definitions, computational complexity, and parameter sensitivity analysis.

All were described in the new subsec. 5.2.:
Regarding the computational complexity. Here is what we think:

PCU simply integrates one or more common supervised and unsupervised algorithms to undersample the training set. The computational complexity is tied to the specific algorithms used, with their exact complexity functions typically outside the scope of this work and usually obtainable from the foundational algorithm papers.

In general, undersampling methods may require time to reduce the number of samples, but they offer clear advantages during model building compared with oversampling techniques.

Comparative Evaluation

While the paper includes comparisons with several resampling methods, it would strengthen the work to include statistical significance tests (e.g., t-test or Wilcoxon) across repeated runs.

Five repeated runs with different splits were reported to ensure that the results are not occurred by chance. Table 2 was modified to include various runs results.

‘*Noise versions were tested using different train/test splits ….’*

Please clarify whether the same random seed or data splits were used across all methods to ensure fairness.

This is illustrated in:
‘*All models use the same training set, test set* ….’

Generalizability

The petroleum dataset is well described, but the method’s generalizability beyond this specific application (e.g., finance, healthcare, cybersecurity) should be better discussed. Consider adding one more benchmark dataset from a different domain to show robustness.

Incorporating an additional industry dataset would be very useful but also would greatly expand the paper beyond our page limit. We have outlined our expectations regarding other domains where PCU may be beneficial within the paragraph:
‘*Our experiments demonstrate the effectiveness of …’*
Additionally, A benchmark dataset was experimented and added to enhance limitation understanding:

*‘Another limitation is that PCU is built on the assumption—that the dataset’*

Figures and Visualizations

Figures 1–4 are valuable but require higher resolution and clearer legends.

Modified all, Thank you.

The “Graphical Abstract” mentioned in the text is missing from the submission. Please include it or remove all references to it.

We apologize for the inaccessibility. It was in the original draft at the bottom of the manuscript; we moved its placement after the abstract, since the figure is typically uploaded separately in editorial managers.

Ablation Study

Since PCU combines unsupervised clustering and supervised refinement, it is important to show the contribution of each step individually (e.g., clustering-only, supervised-only, and combined).

The contribution of the unsupervised component is illustrated using both Sankey diagrams and cluster inclusion tables. And

*‘displayed from right to left ….’*

In figure 4. Also displayed as

*‘green and violet boundaries …’*

In the Graphical abstract.

The performance of the supervised component is reported in the petroleum dataset evaluation, without resampling (Figure 5) and in the Non-resampled results (Table 2). The combined model (i.e., PCU) is evaluated under the ‘Fully-guided’ and ‘Semi-guided’ in both the corresponding figure and table.

Language and Structure

Some sections (e.g., Introduction and Related Work) are overly descriptive. Please focus more on critical comparisons and concise synthesis.

We summarized some, and removed others (marked by ~~strikethrough~~).

Check for consistency in reference formatting (e.g., DOI style and numbering).
Modified, Thank you.

Minor Comments

Typographical and formatting errors occur in several places (e.g., “undersam-pling,” “pos-itive”). Please revise carefully.

Hyphenation settings at lines ends can be modified according to the journal settings.

Include clear variable definitions when first introduced in equations or pseudocode.

Clearer definitions were added specifically in the Algorithm definition (pseudocode). Its placement was moved ahead to Subsec. 5.2, to leverage the definitions from Section 4—such as cluster inclusion, the number of removals, and the rupture stopping point.

The term “Semi-Guided” and “Fully-Guided” branches should be formally defined and consistently referenced.
*‘The Semi-Guided Branch involves training* ..’

Ensure all URLs in the references are correctly formatted and accessible.

Done, thank you.

Clarify whether “noise” in the Two Moons dataset refers to label noise or feature noise.

The Two Moons dataset includes both types. Additionally, we added experiments that vary each type individually. Table 2 presents more test variations in label noise, while Figure 7 was added to evaluate variations in feature noise.

*‘Similar experiment was conducted where the noise’*

We appreciate your time and consideration, and we hope that the revised version meets the expectations of the reviewers and the editorial board
