# Supplementary figures and images for: Correction: Rare event detection by progressive clustering undersampling
Source: PLoS One. 2026 Jun 22;21(6):e0352210. doi: 10.1371/journal.pone.0352210 (PMC13286204; doi:10.1371/journal.pone.0352210)

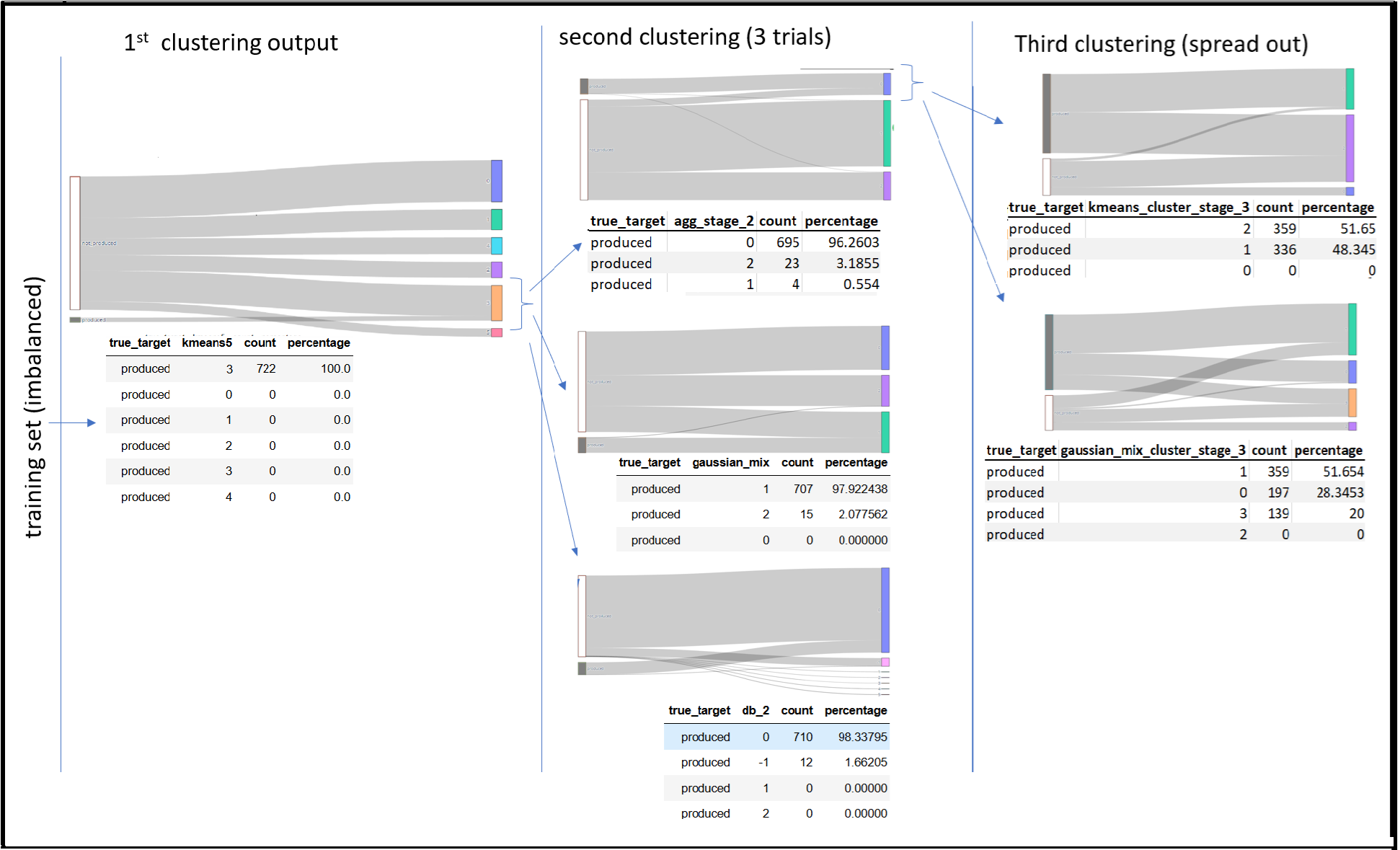

Supplement: S1 Fig — The final result doesn’t change much. (PNG) [file pone.0352210.s001.png]

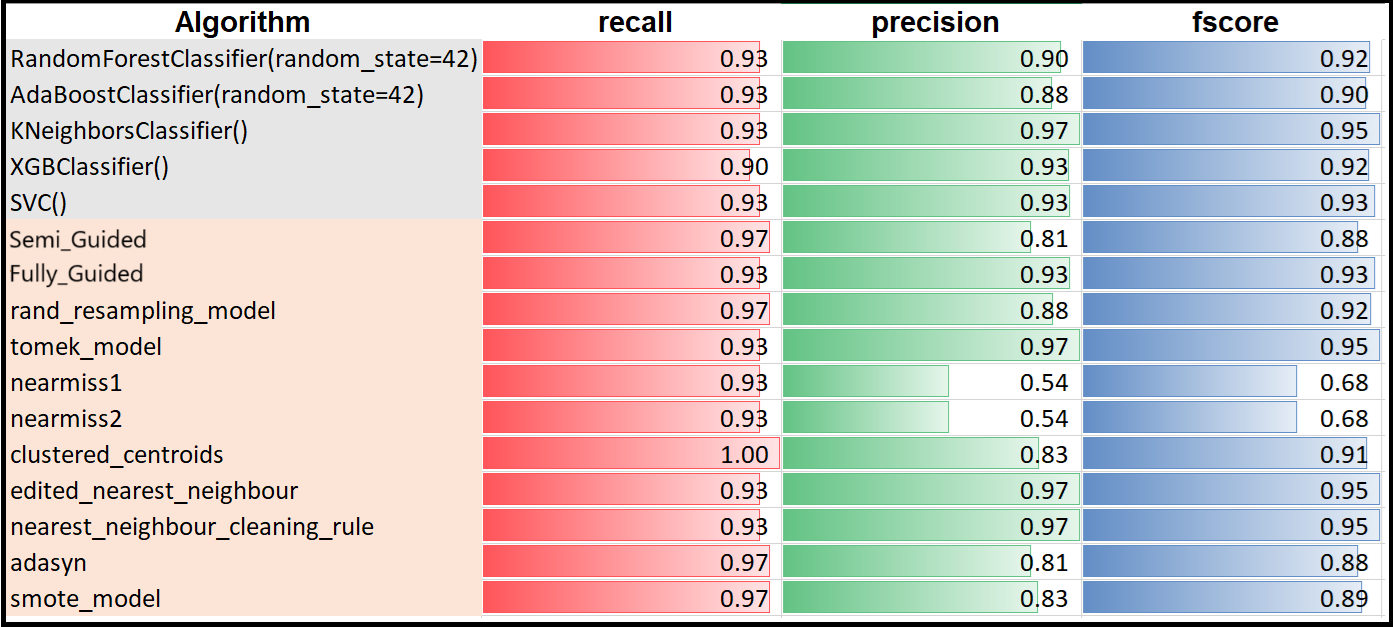

Supplement: S2 Fig — Grey models are supervised classifiers trained directly without resampling. Orange models represent KNN executed after different resampling methods. KNN without resampling is sufficient to achieve the desired results for a simple dataset. (PNG) [file pone.0352210.s002.png]

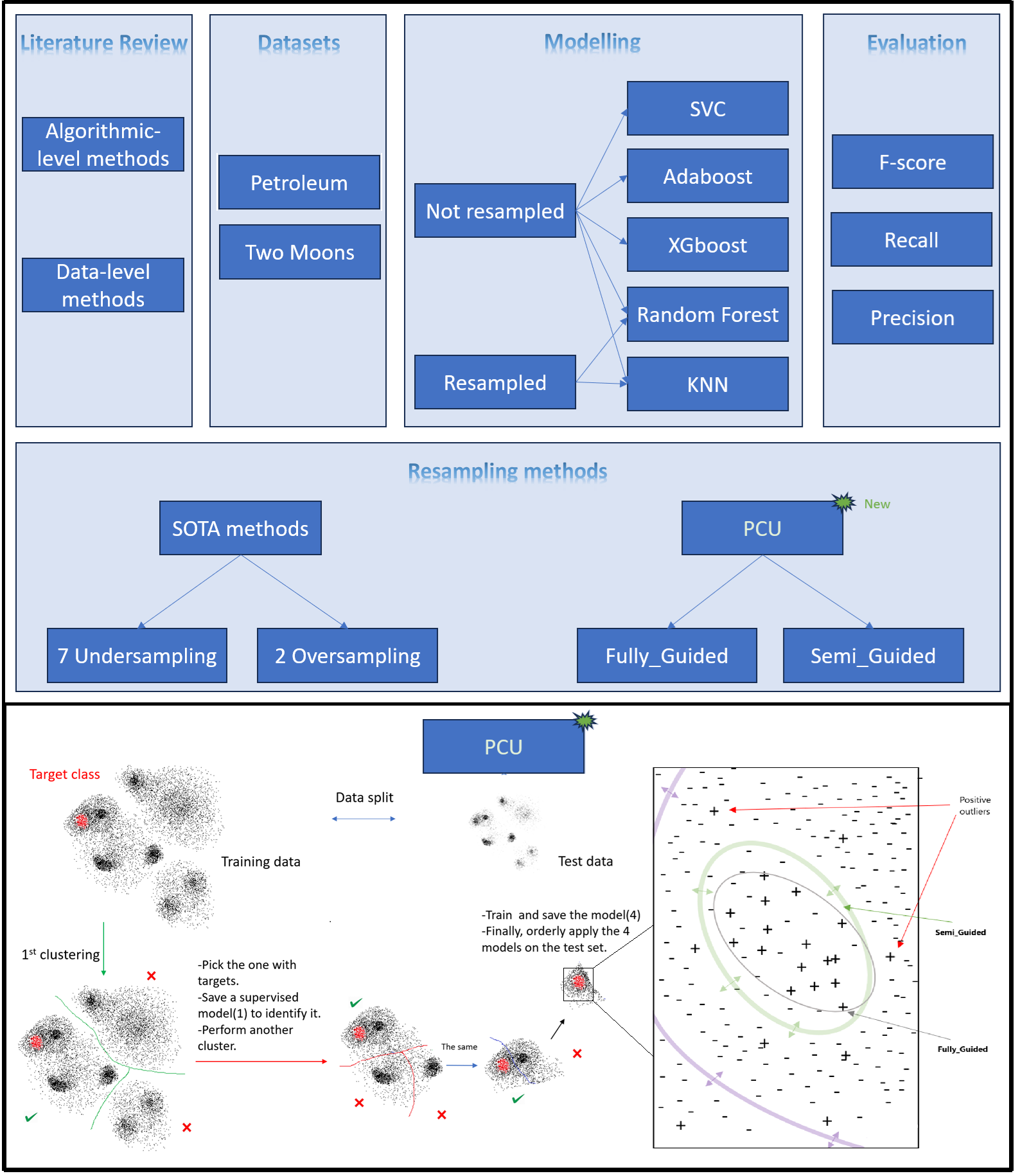

Supplement: S3 Fig — SOTA resampling methods, along with the newly introduced PCU, were evaluated on multiple datasets. The PCU workflow integrates clustering alternated with classification steps to produce Semi-Guided and Fully-Guided decision boundaries. (PNG) [file pone.0352210.s003.png]
